# Enhancing Early Language Disorder Detection in Preschools: Evaluation and Future Directions for the Gades Platform

**DOI:** 10.2196/60424

**Published:** 2025-03-14

**Authors:** María Dolón-Poza, Ana-Marta Gabaldón-Pérez, Santiago Berrezueta-Guzman, David López Gracia, María-Luisa Martín-Ruiz, Iván Pau De La Cruz

**Affiliations:** 1 Grupo de Investigación Innovación Tecnológica para las Personas (InnoTep) Departamento de Ingeniería Telemática y Electrónica, ETSIS Telecomunicación, Campus Sur Universidad Politécnica de Madrid Madrid Spain; 2 Technical University of Munich Heilbronn Germany

**Keywords:** developmental language disorder, simple language delay, adaptive screening system, early childhood education, pervasive therapy

## Abstract

**Background:**

Language acquisition is a critical developmental milestone, with notable variability during the first 4 years of life. Developmental language disorder (DLD) often overlaps with other neurodevelopmental disorders or simple language delay (SLD), making early detection challenging, especially for primary caregivers.

**Objective:**

We aimed to evaluate the effectiveness of the Gades platform, an adaptive screening tool that enables preschool teachers to identify potential language disorders without direct support from nursery school language therapists (NSLTs).

**Methods:**

The study took place in a nursery school and an early childhood educational and psychopedagogical center in Madrid, Spain, involving 218 children aged 6 to 36 months, 24 preschool teachers, and 2 NSLTs. Initially, NSLTs conducted informational sessions to familiarize teachers with DLDs and how to identify them. Following this, the teachers used the Gades platform to conduct language screenings independently, without ongoing support from NSLTs. The Gades platform was enhanced to collect detailed profiles of each child and implemented an adaptive screening model tailored to account for variability in language development. This setup allowed preschool teachers, who are not language experts, to observe and assess language development effectively in natural, unsupervised educational environments. The study assessed the platform’s utility in guiding teachers through these observations and its effectiveness in such settings.

**Results:**

Gades identified language difficulties in 19.7% (43/218) of the children, with a higher prevalence in boys (29/218, 13.3%) than in girls (14/218, 6.4%). These challenges were most frequently observed in children aged 15 to 27 months. The platform demonstrated a high accuracy rate of 97.41%, with evaluators largely agreeing with its recommendations. Teachers also found Gades to be user friendly and a valuable tool for supporting language development observations in everyday educational settings.

**Conclusions:**

Gades demonstrates potential as a reliable and accessible tool for early detection of language disorders, empowering educators to identify DLD and SLD in the absence of NSLTs. However, further refinement of the platform is required to effectively differentiate between DLD and SLD. By integrating Gades into routine preschool assessments, educators can facilitate timely interventions, bridging gaps in early childhood education and therapy.

**Trial Registration:**

Pan-African Clinical Trial Registry (PACTR) PACTR202210657553944; https://pactr.samrc.ac.za/TrialDisplay.aspx?TrialID=24051

## Introduction

### Screening and Prevalence of Developmental Language Disorder

Communication plays a fundamental role in children’s cognitive development. Through communication, children acquire knowledge, express their thoughts and emotions, develop their cognitive ability, and establish relationships with others. Adequate communication development enables them to learn and participate in various social contexts. Therefore, language acquisition during childhood is one of the most critical developmental milestones but with the most substantial interindividual variability in the first 4 years of life. Children start consolidating their language learning process from the age of 4 to 6 years [1-7].

The *Diagnostic and Statistical Manual of Mental Disorders, Fifth Edition* (*DSM-5*), published by the American Psychiatric Association, introduced a new category known as neurodevelopmental disorders (NDDs) that groups different conditions that emerge early in child development before starting elementary school. These conditions eventually persist throughout adulthood [8-12].

The NDDs include communication disorders related to language, speech, and communication impairments. Developmental language disorder (DLD) is classified as a communication disorder. Children with DLD experience challenges in the acquisition and use of language. Language proficiency depends on receptive and expressive abilities. Therefore, children diagnosed with DLD encounter difficulties in both domains [12]. Thus, vocabulary is more limited than expected, and sentences are shorter and less complex with grammatical errors or with speech alterations in narration and comprehension or production of sentences. In addition, they may appear shy or prefer to communicate only with their family members. Children with DLD not only have symptomatology in communication skills but also have difficulties in cognitive and sociocultural processes [5,13-16].

Moreover, a family history of language disorders is often present, and DLD occurs with other disorders, such as autism spectrum disorder and attention-deficit/hyperactivity disorder (ADHD), in 30% of cases [5,17,18]. This makes early detection of DLD in these children more challenging for primary care pediatricians due to the significant comorbidity and similar symptoms with other NDDs [19].

On the other hand, early detection of DLD can be masked by misidentifying it as simple language delay (SLD). DLD is a persistent disorder with a slow rate of improvement and significant variability. This differentiates it from SLD [1,5,20]. However, this indicator does not allow for early detection of DLD, as we cannot identify whether the child has a one-off delay or is showing symptoms of DLD. To differentiate among them, difficulties must be identified in other areas, such as semantics, pragmatics, morphosyntax, and phonology [3]. Therefore, language difficulties manifest through the aforementioned skills that are measurably below what is expected for age, significantly impacting academic achievement, work performance, communication, and socialization [8-11].

DLD is diagnosed at the age of 4 years and is usually stable over time, as interindividual differences in language ability are reduced [1]. However, there may be warning signs that manifest themselves earlier. A study carried out by Vall d’Hebron Hospital Universitari in Barcelona, Spain, highlights a significant portion of children aged 5 to 17 years who exhibit clear symptoms of NDDs but have not been previously diagnosed with DLD [21]. Moreover, communication disorders are one of the significant NDDs with more prevalence in Spanish schools (1.05%-3.42%), along with ADHD and learning disorders [21,22]. Other studies estimated that the population prevalence of language disorders, without any relation to other intellectual disabilities, is 7.58% for children aged 4 to 5 years [17] and 6.4% at the age of 10 years [23]. To summarize, within a preschool setting of 30 children, it can be anticipated that about 2 children may exhibit DLD manifestations [17]. In total, 10% of preschool children present difficulties in language acquisition, of whom 5% to 7% end up being diagnosed with DLD, and the rest are diagnosed with SLD [5].

Before the detection and diagnosis of DLD, there is a prevention phase. Here, the nursery school plays a key role, as it is within this environment that the child spends most of their time and is involved in different interactions [1]. These interactions contribute to developing their communicative skills (communicative intention, nonverbal communication, imitation, waiting, etc) with peers of a similar age and teachers. Research has demonstrated a correlation between children with DLD and their academic performance. In fact, 88% of these children fail to meet the necessary curricular standards during their first year of school [14,17]. Hence, there is a pressing need to support education professionals with enough training and knowledge to identify early indicators of potential language disorders [1,24-27].

In the detection phase, the aim is to identify early whether a child is suspected of having some NDDs. To achieve adequate detection, tools such as screening forms and questionnaires allow information to be compared with previously defined risk indicators [20,28-32]. Some regions, such as Madrid (Spain), have early childhood educational and psychopedagogical guidance teams supporting nursery schools’ detection phase. These teams engage in preventive actions and collaborate to detect developmental issues during the initial years of a child’s life. If an educational case is identified, it is referred to the early intervention centers responsible for the assessment and intervention phase [33].

The assessment and intervention cover different aspects. During the assessment phase, it is necessary to analyze the family environment through interviews with relatives. In addition, hearing tests should be administered to eliminate potential associated psychological disorders. Evaluation of communication, playing with peers, comprehension, language production, and psychomotor skills are also essential. Finally, this information is correlated with medical history [6,14,15,20]. The intervention phase for DLD should be multidisciplinary, involving teachers, occupational therapists, speech therapists, and psychologists skilled in children’s language development techniques [7,24,27,34].

### Gades: A Screening Tool for DLD

Gades functions as a clinical decision support system designed to grant expert knowledge regarding language disorders to preschool teachers. It offers a screening form to assess the language acquisition process during the early stages of a child’s development. Gades allows the implementation of screening systems in natural environments for the child, thereby embracing the principles of pervasive therapy [35].

Pervasive or ubiquitous therapy is a therapeutic approach supervised by professionals that aims to promote engagement in activities focused on improving the individual’s life within their natural environment through the support of information and communication technologies. It aims to be person centered instead of focusing on a specific clinical pathology. It especially seeks to identify how a problem impacts and manifests across various contexts [18,36]. This approach has the same phases of prevention, detection, assessment, and intervention as normative therapy. Moreover, using information and communication technologies ensures consistency and continuity of the therapeutic process within the person’s natural setting [32,37-39].

For children’s pervasive therapy, its application extends beyond the clinical center to environments like the child’s school or home. Nursery schools are included in these settings. Being places where children spend much of their time, they are crucial for the early detection of DLD [24]. During this educational phase, teachers or educators and specialists in therapeutic pedagogy, such as nursery school language therapists (NSLTs), are typically involved. However, many nursery schools lack NSLTs, and even when they are available, fewer than half of the children suspected of having a language disorder are referred to or receive support from these specialists [1,7,17,23,27].

Martín Ruiz et al [35] developed and evaluated a previous version of Gades. The platform’s cornerstone is a knowledge base (KB) that includes 106 milestones related to children’s language acquisition based on age, defined and agreed upon by language experts. The previous version of this KB covered children aged 1 month to 72 months [35]. Building upon the findings of the previous experiment, increased instances of suspected DLD were identified in children aged 0 to 3 years. Consequently, in the current experiment, we focus on prioritizing the implementation of Gades during this developmental period. To extend this goal, Gades has undergone modifications and enhancements to its functionalities to gather additional pertinent information concerning language acquisition in children. These adaptations include integrating inquiries specific to the bilingual context of the children and their family backgrounds and evaluating results from the Gades across different language areas.

When using Gades, the corresponding milestones, tailored to the child’s condition, are incorporated into a screening form through questions that education professionals use to assess a child’s language development level. The milestones are categorized into 2 main types: “warning milestones,” which prompt a reevaluation, and “alarm milestones,” indicating the need for direct referral to health professionals. Gades’ KB assesses 4 areas of speech and language development: sensory reception, speech perception, production, and pragmatic.

After the form adapted to the child is completed, Gades generates an evaluation result, including a suggestion for action if deviations from typical child development stages are detected. Therefore, it provides education professionals with expert knowledge on language acquisition, enhancing the early detection of DLD. Furthermore, this screening form acts as a psychopedagogical report for early intervention health professionals. Gades can aid in identifying the language development profile of a child suspected of having DLD through the assessment of processing-oriented and performance-based tasks.

**Figure 1:**
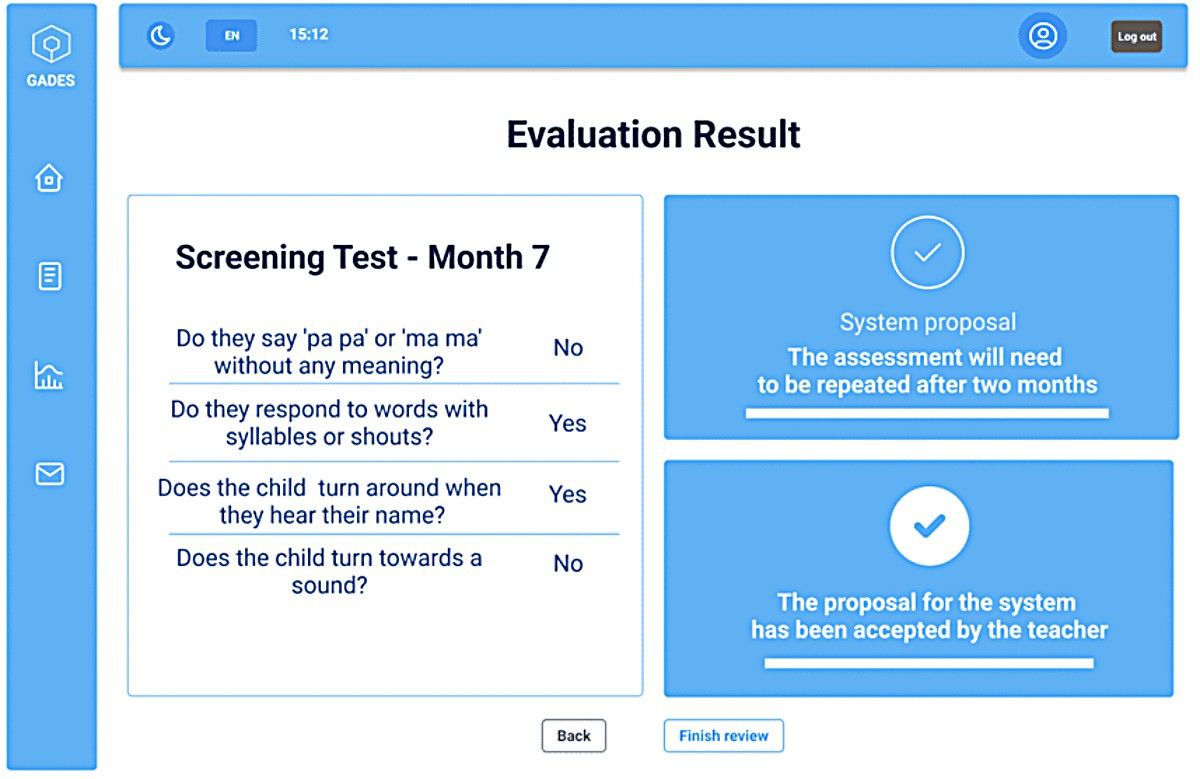
Gades' evaluation result page for a child aged 7 months. In total, 4 questions were asked based on the child’s age, and the system suggested repeating the evaluation in 2 months. The preschool teacher concurred with the system’s recommendation.

Figure 1 depicts the graphical user interface of Gades, showcasing the evaluation results of consultation for a child aged 7 months. The platform displays the outcomes of the screening form, including the posed questions and the teacher’s responses. Response options include “yes” if the child performs the specified action, “no” if not, and “do not know/no answer” for unknown answers. This form is completed based on the preschool teacher’s observations of the child without direct interaction. After review, the system recommends that the assessment be repeated in 2 months, a suggestion that the overseeing teacher has validated. Gades also features additional tools for child registration, language evaluation, and user profile management.

### Goal of This Study

For effective early detection and intervention of DLD, it is essential to offer sufficient services to these children to minimize the impact on their social, emotional, and educational development. Thus, language disorders often require a multidisciplinary approach among professionals from diverse fields such as education, speech-language therapy, and medicine to extend their reach into various settings [17,18,24]. Supporting training programs and systems for health and education professionals fosters collaboration. This collaboration facilitates the creation of adequate evaluation and intervention plans that meet the diverse needs of individuals with language difficulties, enhancing the quality of care and inclusivity [22,26,30,31,38,40].

Within this collaboration, the early detection of warning signs, which are potential indicators of language disorders in children, should be an integral part of the daily work of those interacting with children [1,7]. Supporting early childhood education professionals is crucial to ensure that they possess adequate knowledge and tools for preventing and detecting early language development issues in children aged 0 to 3 years because behaviors and warning signs related to language disorders can be identified from the age of 2 years onward [1,3,4,24]. It facilitates appropriate early diagnosis of DLD or SLD through subsequent assessment at early intervention centers. Moreover, given the absence of an NSLT in some schools, preschool teachers must have access to these resources.

Considering the higher incidence of suspected DLD observed in children aged 0 to 36 months in the previous experiment [17,23], this paper aims to emphasize the use of Gades during this period, targeting kindergarteners aged 6 to 36 months in a nursery school and an early childhood educational and psychopedagogical center. Gades has been adapted and expanded to gather more relevant information on language acquisition, including questions tailored to bilingual contexts and family backgrounds. Therefore, our primary goal is to evaluate Gades’ ability to differentiate between typical and atypical language development for the target ages with these minor adjustments.

As the previous experiment consisted of a controlled environment, this paper also presents the relationship between preschool teachers and the Gades platform. Specifically, how Gades serves as a valuable tool for guiding nonexperts, such as preschool teachers, in conducting language observations. Therefore, it evaluates Gades’ effectiveness in a real setting and its adherence to clinical criteria without direct supervision or intervention of language experts or technical staff. A potential challenge is also associated with integrating these technological tools into unsupervised educational settings to support therapeutic interventions [41].

Moreover, this screening process aims to identify the patterns indicative of a child with problems in language development, including SLD or DLD. Considering the updated *DSM-5* criteria, language development in children occurs within heterogeneous and dynamic contexts. Hence, it is imperative to use screening tools to identify significant patterns and milestones in the language development of a child with DLD, facilitating early detection and intervention. To accomplish this, the screening tools must be intersectional, considering influential parameters, such as clinical conditions and the sociocultural and linguistic environment surrounding the child. Consequently, the final goal is to outline the framework for an adaptive screening model for language disorders that reflects the child’s current developmental and social context. This approach ensures a comprehensive and nuanced understanding of each child’s unique situation, enhancing the accuracy and effectiveness of early detection methods. Therefore, this study hypothesizes that the Gades platform can effectively support preschool teachers in the early detection of language development difficulties, including DLD and SLD, within natural educational settings, achieving high accuracy rates and providing actionable insights without requiring direct supervision from language disorder specialists.

## Methods

### Overview

This study used a prospective observational design to evaluate the updated Gades platform in real-world educational settings. The design involved monitoring children aged 6 to 36 months and their evaluators—preschool teachers and early childhood educators—over a defined period, collecting data as they naturally occurred during regular classroom activities. This approach was selected to ensure that the platform’s performance could be assessed in authentic, uncontrolled conditions, reflecting its practical application. By focusing on how educators integrated Gades into their daily routines, the study aimed to capture unbiased insights into its usability and effectiveness in identifying early signs of language development difficulties.

### Requirement Analysis

Individuals encounter varied communicative situations involving diverse entities and methods in everyday life. Current diagnoses and treatments for various disorders frequently overlook key characteristics, such as diversity, heterogeneity, and the role of socialization. The *DSM-5* and numerous studies highlight the necessity to consider interindividual differences in language acquisition early on. These sources suggest that assessments of language, speech, and communication should consider sociocultural aspects, linguistic context (including dialects), and socioeconomic status, as these aspects directly influence language development [1,12,16,17,19,21,31,32,42-44].

Bosch et al [21] demonstrated that 1.05% of the participants had communication disorders, with the most significant factors being foreign origin, genre, socioeconomic status, and age. Consequently, the diagnosis of language impairment should consider the individual’s background, bilingual context, direct clinical observations in settings such as home or school, and results of standardized tests to assess the severity of the disorder.

### The New Gades Platform

Building on the previous experiment with the Gades platform [35], we updated it with new technologies and features. These enhancements enable the collection of additional information from children to conduct more accurate and suitable assessments of a child’s language development. This includes gathering details about the child’s family background, linguistic environment, and medical history, all of which contribute to a more comprehensive understanding of each child’s unique developmental context. Therefore, incorporating these minor adjustments delineates the structure of an adaptable screening model that considers the variability in language development. This methodology guarantees a thorough and nuanced comprehension of each child’s individual circumstances, enhancing the precision and efficacy of techniques for identifying potential issues in language development.

Implementing Neo4j (Neo4j Inc), a graph database technology, is crucial in optimizing our Gades platform, specifically in enhancing the accuracy of language disorder detection. Neo4j allows for a more natural and flexible representation of complex data and interconnected relationships between them, which is essential for modeling the intricate interactions and dependencies observed in child language development [45]. In the context of Gades, Neo4j facilitates the dynamic integration and analysis of large volumes of data related to language development milestones, assessment responses, and individual characteristics of children. For example, the graph structure enables us to directly link children’s responses to specific questions with language development patterns and automatically flag connections that indicate potential delays or deviations from typical development.

**Figure 2:**
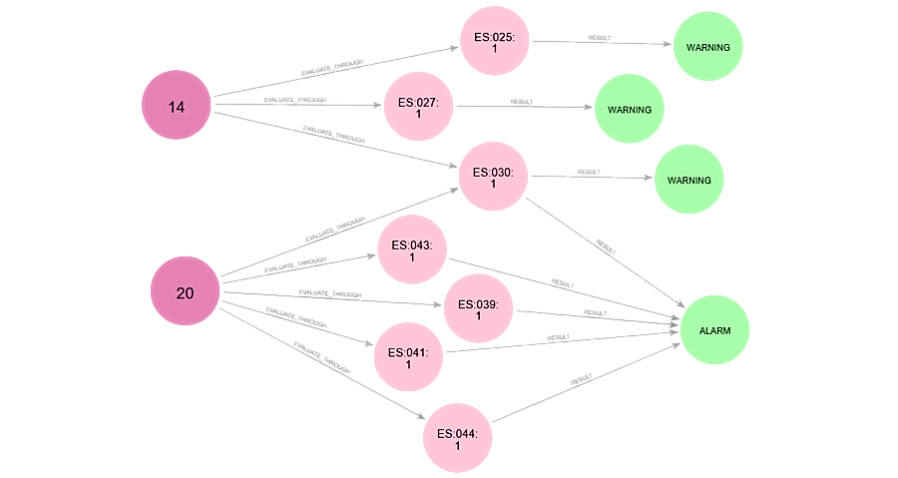
Gades' knowledge base in Neo4j: milestone months (dark pink node), questions identifiers (pale pink nodes), and suggestions (green nodes). Each month has various questions to evaluate language acquisition (for a child aged 14 months or 20 months) with suggestions such as typical development, repeat evaluation, or advising referral to early childhood care services.

Therefore, the KB has also been migrated to Neo4j graph databases. As illustrated in Figure 2, each milestone is represented through a month (dark pink node) with several questions (pale pink nodes) and a final suggestion (green nodes). Thus, in the figure, a dark pink node is represented each month, corresponding to different questions designed to evaluate the child’s language proficiency, depicted by pale pink nodes. Notably, some questions are linked to multiple months, reflecting the dynamic nature of language development assessment. Different action suggestions can be generated based on the answers to these questions, such as confirming typical development, recommending reassessment, or advising referral to early childhood care services (green nodes represent these). This graph-based approach facilitates a nuanced analysis of the child’s language development, allowing for tailored interventions sensitive to each child’s unique progression.

With the updates to Gades, the system has addressed feedback from educators seeking more information on DLD. Previously, questions were categorized by language development areas and were only accessible to the system’s technical staff. However, with the recent improvements, information about various skills is now accessible to the evaluation staff. This improvement means that by using Gades, preschool teachers and other professionals can gain deeper insights into the specific language development areas being assessed. Such transparency not only empowers educators with a better understanding of language development but also enables them to contribute more effectively to detecting warning signs.

Table 1 illustrates the classification and number of questions across different categories. The sensory reception category is administered in the earliest months of life, primarily to assess for hearing issues, and gradually transitions into language perception. This aspect entails the capacity to receive, process, and comprehend linguistic information through sensory channels, whether auditory or visual. Pragmatics includes conversational skills, coherence, cohesion within discourse, and the functional and social use of the language. Finally, language production encompasses phonology, morphosyntax, semantics, and nonverbal communication. This structured approach allows for a comprehensive assessment of a child’s language development, targeting specific areas crucial for early detection and intervention of language disorders.

**Table 1:** Classification of questions according to language category [35].

Language development domains	Number of questions
Sensory reception	3
Language perception	32
Production	48
Pragmatics	25

### Hypothesis and Experiment

We aimed to validate the reliability and effectiveness of the new Gades platform in a real setting without the direct supervision or intervention of language experts or technical staff. We explored the interaction between preschool teachers and the Gades platform, highlighting how Gades functions as a valuable tool for facilitating language observations to detect warning signs of language difficulties by nonexperts. The Gades platform is specifically designed to assist in detecting early language difficulties that may signal potential DLD. By focusing on these early warning signs, Gades supports the early detection and monitoring of language development challenges in real-world educational settings.

The Gades KB was established in a previous study [35] and is grounded in standardized tests commonly used to assess DLD. This ensures that Gades relies on validated methodologies to identify and interpret potential language difficulties.

The study’s population sample consisted of 218 children aged 6 to 36 months from 2 educational centers in the city of Madrid: a preschool center and an early childhood educational and psychopedagogical center. All children enrolled in the first cycle of early childhood education, which aligns with this age range, participated in the study. This selection was based on recent evidence highlighting the critical importance of identifying language difficulties during this developmental period [1,3,4,24]. The screening data were collected from March to June 2023, providing an initial assessment of Gades’ effectiveness in a real-world educational setting. Notably, access was limited to Spanish-speaking and some bilingual children.

The screening assessments for DLD involved the active participation of 24 nursery school educators and preschool teachers and a clinical professional from the early childhood center. Throughout this study, we will refer to them as evaluators. Hence, 2 categories of evaluators were involved in the experiment. The initial cohort consisted of professionals from early childhood centers, who, while not specialized in DLD, possessed adequate knowledge of NDDs. The second group comprised nursery school educators and preschool teachers, who may lack specialized language expertise but possess valuable insights into the children’s competencies under their care. Before starting the experiment, the evaluators received training from qualified professionals affiliated with the Specific Language Disorder Association of Madrid. This training aimed to deepen their understanding and expertise regarding DLD and its identification with children aged 6 to 36 months. The training, delivered in a concise 2-hour format, was designed to ensure efficiency while providing comprehensive insights. By the session’s conclusion, it was anticipated that the educators would have developed a more nuanced understanding of typical language milestones and a heightened capacity to discern early indications of language difficulties in children under their care.

During the evaluations, they operated without direct assistance from the training professionals or the Gades technical support team. Consequently, the experiment was conducted in an uncontrolled setting, relying exclusively on the evaluators’ direct observation of the children’s behavior for further evaluation through Gades. The evaluations were conducted following the guidelines recommended by Gades, aiming to ensure that the process was as effective and informative as possible within the constraints of the study’s design.

**Figure 3:**
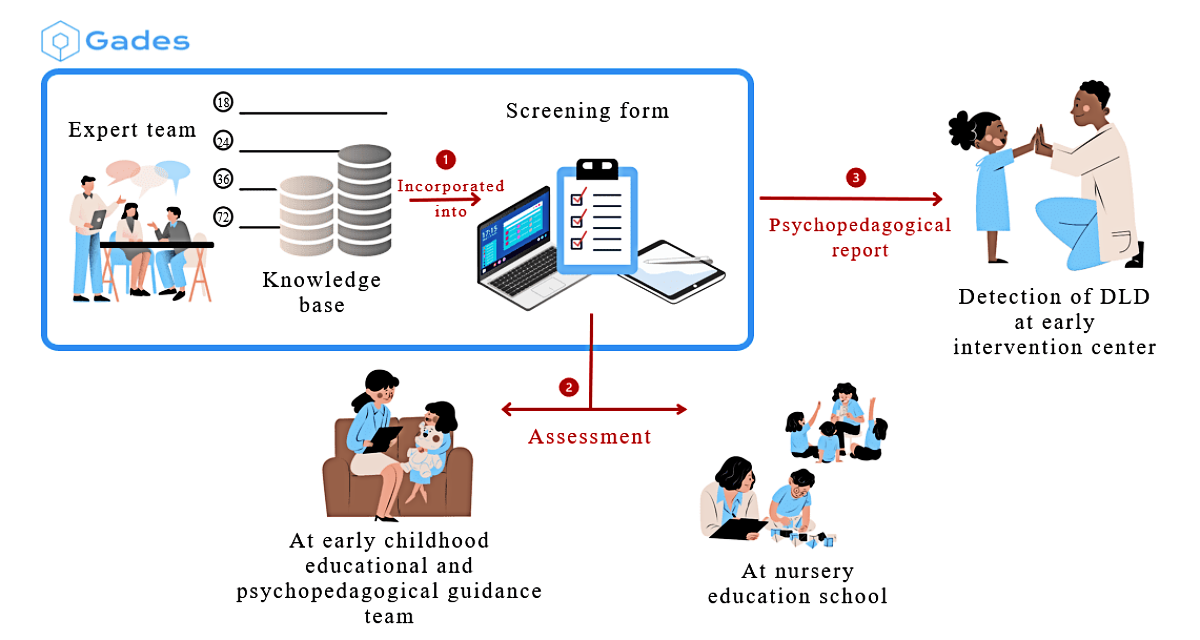
Gades’ detailed framework. The process starts with expert validation of the knowledge base, integrated into a screening form with control and language questions. Evaluators assessed the children, and Gades provided recommendations, such as reassessment or referral, forming the basis of a psychopedagogical report. DLD: developmental language disorder.

Figure 3 outlines the Gades’ framework, starting with an initial phase where a series of meetings were conducted to review and validate the KB established in the previous experiment. This phase involved experts in language disorders and the technical personnel from Gades to ensure the KB’s relevance and accuracy. The panel of experts, who were also involved in constructing the KB as part of a previous study [35], recommended that including 3 to 6 questions per month would constitute an adequate measure for detecting language development issues. In the context of this study, the same experts conducted a review and confirmed the continued validity and applicability of the content of the KB. Once the KB was validated, the second phase, the screening process, began.

The assessments were carried out by experienced female educators and professionals proficient in using digital platforms and information technologies. They used mobile devices and workplace computers to conduct evaluations, strategically scheduling them during the children’s nap time around midday to minimize distractions. This approach involved each evaluator conducting assessments of the children under her care, which consisted of 2 parts. The first part included control questions related to the child’s medical history and linguistic context, serving as a baseline for understanding each child’s unique background. The second part consisted of the language difficulties screening questions. These questions were meticulously developed in collaboration with experts to cover risk parameters for the early detection of DLD.

After the screening form was completed, the Gades system generated a recommendation to the evaluator, such as suggesting a repeat assessment in the upcoming months or indicating the need for a referral to an early intervention center. If a referral was recommended, nursery schools in Madrid forwarded the result to the expert through a psychopedagogical report. The designated expert, a language professional, was responsible for conducting a standardized evaluation tailored to the child’s needs. Meanwhile, the educator played a supportive role by closely observing the child’s progress in class and paying particular attention to areas of difficulty. Regular communication between schools and the expert was maintained with biweekly visits to ensure follow-up and support.

The application of the Gades platform was deliberately structured to be as nonintrusive as possible, allowing evaluators to focus on their primary duties while still contributing to the study. On average, each child underwent 1.24 (SD 0.49) assessments, indicating a consistent approach to the number of evaluations. The average duration of an assessment was 70 (SD 41.8) seconds per child, demonstrating the platform’s efficiency in collecting data within a brief timeframe. To validate the Gades platform, both quantitative and qualitative analyses were conducted.

Quantitative validation focused on assessing the platform’s accuracy and reliability. Descriptive statistics were used to summarize the duration and frequency of assessments. Agreement between the system’s recommendations and evaluators’ decisions was measured using concordance rates, calculated as the proportion of evaluations where the evaluators accepted Gades’ recommendations. Statistical significance was set at *P*<.05. In addition, the platform’s accuracy rate (97.41%) was determined by dividing the number of accepted suggestions (n=263) by the total number of evaluations (N=270). Instances of disagreement were analyzed qualitatively to identify patterns or recurring issues.

Qualitative validation focused on the integration of Gades into educational settings and its usability as perceived by preschool teachers. Evaluators were asked to provide feedback via a standardized questionnaire, following a validated system usability scale [41]. Responses were anonymized and collected outside the Gades platform to reduce potential bias. Evaluators also provided open-ended feedback to capture their experiences and insights, highlighting the platform’s strengths and areas for improvement. The qualitative data were analyzed thematically to identify trends and recurring themes.

This combination of statistical and thematic analyses ensured a comprehensive validation of Gades. By comparing the platform’s outcomes against educator feedback and established clinical criteria, the study aimed to validate Gades’ ability to aid in the early detection of language development issues, such as SLD or DLD. Furthermore, comparisons between the initial version of Gades (2014) [35] and the current version highlighted improvements in adherence to clinical standards and system usability.

### Ethical Considerations

This study, including the validation of the Gades platform and the experiment conducted through it, received ethics approval from the Ethics Committee of the Polytechnic University of Madrid in October 2023 GYGSDAALTD-MLMR-DATOS-20231010 and GYGSDAALTD-MLMR-HUMANOS-20231010). This approval ensures that both the use of the Gades platform and the specific experimental protocol comply with ethical standards, including data protection, participant rights, and research transparency.

For this study, an opt-out consent model was implemented. Participants were informed in advance about the study’s objectives, the nature of the data collection, and the measures taken to ensure anonymity. They were given the opportunity to decline participation or withdraw their data at any point without providing a justification. Consent was implied by the participants’ choice to continue with the evaluations, as no identifiable personal data were collected. Anonymized data were securely provided to the research team.

The professionals overseeing the evaluations maintained confidentiality in accordance with professional secrecy standards. Only the principal investigator had access to the evaluation results, which were securely stored in an encrypted database, accessible solely through authorized credentials.

## Results

### Sample Summary

The sample size for this study was calculated using a finite population correction formula to ensure adequate representation and statistical power. On the basis of demographic data from Madrid, the estimated population of children aged 6 to 36 months was approximately 150,000 [46]. With an assumed prevalence of DLD of 7%—a conservative estimate derived from epidemiological studies [17]—a 95% CI (*Z*=1.96), and a margin of error of 5%, the required sample size was calculated. The formula incorporates the finite population adjustment to account for the limited size of the target population, yielding a minimum required sample size of approximately 100 participants. This calculation also considered the variability in language development within the population.

The study’s population sample comprised 218 Spanish-speaking children aged 6 to 36 months, more than double the calculated minimum sample size, ensuring robust statistical power and enhancing the reliability of the results. It featured a control group of 134 children from the nursery school (center 1) without any previous diagnosis or visible language difficulties and an experimental group of 84 children from the early childhood educational and psychopedagogical center (center 2), suspected of NDDs. Both groups were matched by gender to maintain homogeneity. Each child was assigned a random identifier for data anonymization and to facilitate subsequent analysis by the research team.

In Madrid’s nursery schools, for each child, the evaluations were conducted by 2 assigned preschool teachers, also called an “educational pair.” The concept of an “educational pair” refers to 2 professionals sharing responsibilities and collaborating for the children’s development and well-being, enabling the distribution of evaluation tasks between them. Therefore, in classrooms with children aged <12 months, each teacher evaluated an average of 4 (SD 0) children. For groups with children aged from 13 to 24 months, the responsibility increased to an average of 6.75 (SD 0.577) children per teacher. For those aged >24 months in the classroom, the average was 5.9 (SD 6.610) children per preschool teacher. Evaluations at the early childhood educational and psychopedagogical center were conducted by the teacher specializing in therapeutic pedagogy (NSLT).

Table 2 presents an overview of the study’s sample, detailing the distribution of the children by age and gender: 45.4% (99/218) of the girls and 54.6% (119/218) of the boys participated. Within this group, 36% (36/99) of the girls and 40.3% (48/119) of the boys came from the early childhood center, initially identified with potential NDD concerns. Specifically, for participants aged >3 years, the study included 4 boys and 2 girls, all aged 37 months, except for 1 girl who was aged 38 months. The second year of the initial preschool cycle had the highest representation, with the largest number of children aged between 18 and 28 months, averaging 10 (SD 3.045) children per month. The average age in months per grade was distributed as follows: up to 12 months, the average age was 10.020 (SD 1.136) months; from 13 to 24 months, the average age was 20.331 (SD 1.108) months; and from 25 to 36 months, the average age was 31.578 (SD 1.094) months.

**Table 2:** Distribution of children by center, gender, and age range^a^.

Grade			Up to 12 mo (n=26), n (%)		13-24 mo (n=102), n (%)		25-36 mo (n=84), n (%)
**Children from** **center 1**							
	Female	10 (38.4)		22 (21.6)		31 (36.9)	
	Male	8 (30.7)		24 (23.5)		39 (46.4)	
**Children from** **center 2**							
	Female	5 (19.2)		22 (21.6)		9 (10.7)	
	Male	3 (11.5)		34 (33.3)		11 (13.1)	

^a^Center 1 is the nursery school, and center 2 is a psychopedagogical center. The age ranges align with the preschool grades. The second year of the initial preschool cycle had the highest representation (102 children).

The screening form’s initial section included questions regarding the child’s medical history and linguistic context. Birth complication cases were more prevalent in center 1, particularly among male participants. Results indicated that 8.2% (11/134) of the children with no previous diagnosis of NDD experienced birth complications, including prenatal, perinatal, and preterm risks. 2.9% (4/134) were reported among females while among males, 5.2% (7/134) were observed. In center 2, only 2% (2/84) cases were reported in males and no cases in females. Notably, 3 children faced more than 2 of these risks, such as family history combined with perinatal risk. The mean gestational weeks remained relatively consistent across both centers and genders, with slightly more variation among girls. The average gestational age was 39.518 (SD 5.363) weeks, female children had an average of 38.984 (SD 2.36) weeks in center 1 and 40 (SD 0) weeks in center 2, while males had 39.563 (SD 1.29) weeks in center 1 and 39.791 weeks (SD 1.44) in center 2. A baby is considered premature if born before the 37th week of pregnancy. According to the study, only 3.7% (8/218) of the children were born preterm, highlighting specific early life factors that could influence developmental outcomes. Instances with a family history or bilingual cases were limited. Among the 218 cases studied, only 2 reported a family history of other NDDs such as autism spectrum disorder. Information regarding the bilingual context was exclusively available for children from center 1 (N=134). 7 (5.2%) cases were found among females, and 3 (2.2%) cases were recorded in males. It suggests that the language development impact of being raised in a bilingual environment could not be thoroughly assessed across the entire sample.

### Overview of the Obtained Health Results

This section presents the results obtained from the evaluations that were conducted. A total of 270 assessments were completed, accounting for instances where children underwent multiple assessments. Retaining only the latest assessment conducted for each child (218 evaluations), there were 19.7% (43/218) children, which resulted in a suggestion to refer them to an early intervention center for a specific assessment by a professional. These types of suggestions were classified as alarms. That was the most severe system decision. Table 3 shows the results of the final assessments. The system has recommended repeating the remaining assessments in subsequent months to further evaluate and discard language issues. Suggestions that indicate repeat evaluation are classified as warning type.

**Table 3:** Distribution of Gades’ final suggestionsa (N=218).

Description	Distribution, n (%)
Typical developmental	117 (53.7)
Refer to early childhood care	43 (19.7)
Repeat assessment (within 3 mo)	28 (12.8)
Repeat assessment (within 2 mo)	19 (8.7)
Repeat assessment (within 1 mo)	11 (5)

^a^More than half of the sample (n=117, 53.7%) were identified as typical language development. However, 19.7% (43/218) of assessments identified language difficulties as indicative of possible simple language delay or developmental language disorder.

Table 4 displays the distribution of assessment types by gender. It reveals that more alarm cases were identified in boys (29/218, 13.3%) compared to girls (14/218, 6.4%). In addition, both genders had a similar frequency of warning assessments and neurotypical results.

**Table 4:** Distribution of evaluations by suggestion type and gender. More alarm cases were identified in boys (29/218, 13.3%) compared with girls (14/218, 6.4%). Both genders had a similar rate of warning assessments and neurotypical results (N=218).

Gender and evaluation			Evaluations, n (%)
**Female**			
	Alarm	14 (6.4)	
	Warning	29 (13.3)	
	No findings	56 (25.9)	
**Male**			
	Alarm	29 (13.3)	
	Warning	29 (13.3)	
	No findings	61 (27.9)	

Gades identified more cases of alarm and warning in children already undergoing evaluation by specialists than from the center (alarm: 29.7%; warning: 32.1%), but we do not know whether these children had language disorders. Hence, among children already experiencing some difficulties, it identified that there may be some indication of language difficulties (ie, DLD or SLD).

Table 5 shows the distribution of evaluations that were classified as an alarm because it was detected that the assessed child did not meet the language acquisition milestones expected for their age. The incidence of language impairment from center 2 was comparable between boys and girls (15:10) and 30% (25/84) of the cases were detected as alarms. Meanwhile, for children without a suspicion of NDD coming from nursery school (center 1), difficulties in language and communication were detected in 13.4% (18/134) of the cases. However, a notable discrepancy was observed between boys and girls (14:4).

**Table 5:** Distributions of alarm evaluations by gender, month, and center^a^.

Month		9	10	11	14	15	16	18	19	20	24	25	26	27	28	30	32	37	Total
**Children center 1**																			
	Female	0	1	1	0	0	0	0	0	1	1	0	0	0	0	0	0	0	4
	Male	0	1	0	1	0	0	4	0	0	1	1	2	1	0	1	1	1	14
	Total	0	2	1	1	0	0	4	0	1	2	1	2	1	0	1	1	1	18
**Children center 2**																			
	Female	1	0	0	0	2	0	1	3	0	0	0	1	1	1	0	0	0	10
	Male	0	0	0	0	3	1	4	2	2	2	1	0	0	0	0	0	0	15
	Total	1	0	0	0	5	1	5	5	2	2	1	1	1	1	0	0	0	25

^a^For children coming from center 1, language issues were detected in 13.4% (18/134) of the cases. The incidence of language issues from center 2 was comparable between male and female participants (15:10), with alarms detected in 30% (25/84) of the cases.

Notably, most of the suspected cases were detected in months 15, 18, 19 and 24. All these months correspond to the second year, encompassing children aged between 1 and 2 years. Upon thorough analysis of the evaluations during these months, difficulties were discerned in the language production category for month 15. Half (23/40, 50%) of the children resulted in alarms. When compared to assessments from previous months, several observations emerged. First, most questions in this category were not been asked in previous months, as the child’s age renders them too young to possess these competencies. Second, only 1 question, “first disyllables (one word in addition to dada and mama)?” was repeated in months 12 and 14, with half (8/14, 57%) of the assessed children answering negatively in both months. For month 15, all respondents answered negatively.

Month 18 had the highest number of detected suspected cases (9/11, 82%). During one of the meetings, educators expressed that they had observed more issues in language production, citing example, such as the child’s inability to articulate certain words. In addition, similar questions asked in previous months also resulted in alarms. For instance, the one with the most error answers was “first bisyllables (two words in addition to dada and mama)?” In subsequent months, specifically month 19, identical pragmatics and language production questions were asked, and negative responses were received.

In month 24, 40% (4/10) of the evaluations raised alarms. Upon analyzing the questions, 3 were related to pragmatics and language comprehension, which were previously asked in earlier months. Most children satisfactorily answered these questions, indicating alignment with their current developmental stage. Therefore, the cases prompting alarms may signify potential instances of children with SLD or DLD.

**Figure 4:**
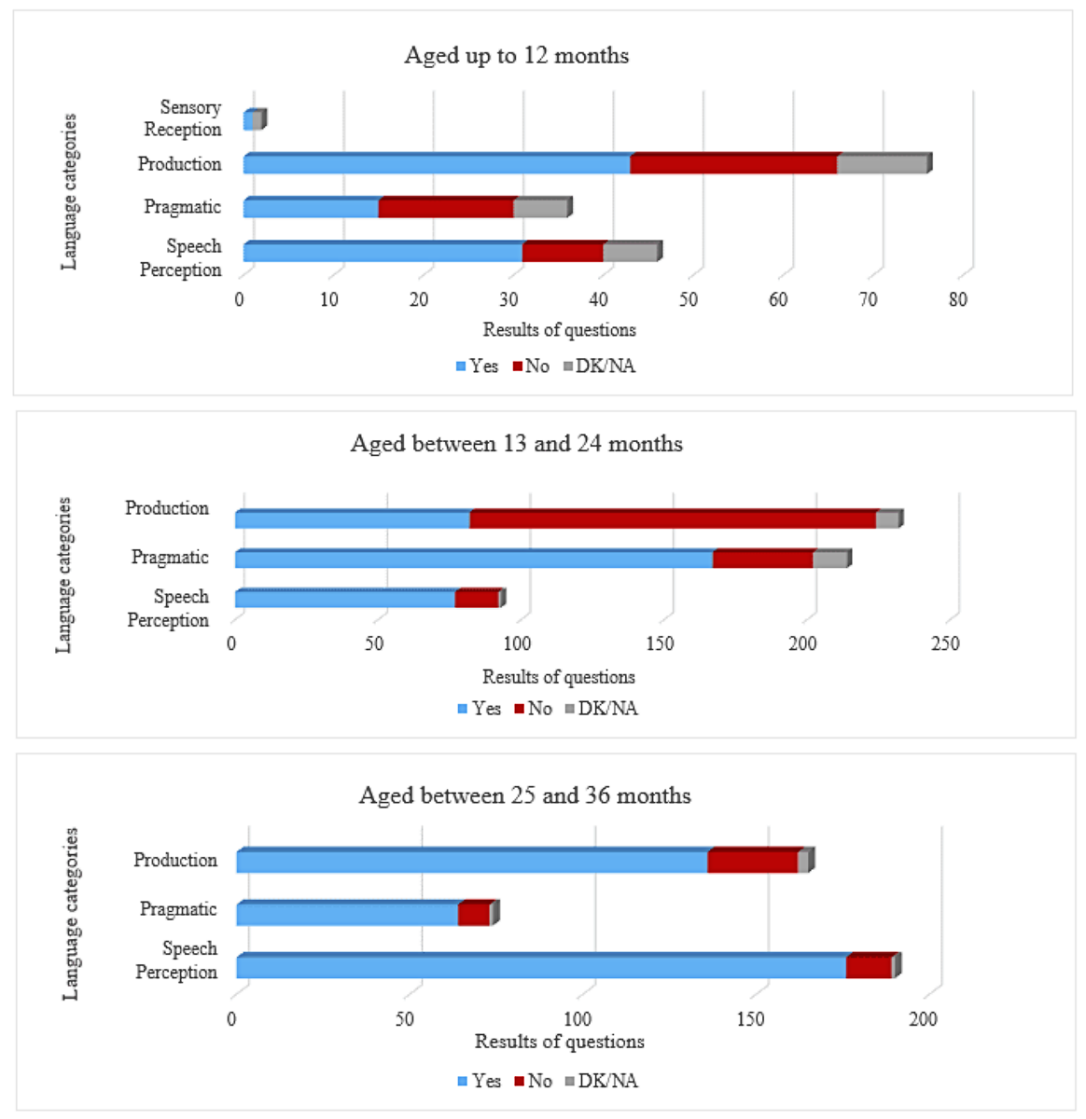
Distribution of results based on language-category questions. Each question is categorized according to language category (refer to Table 1 for more information) and can be answered with a yes, no, or do not know/no answer (DK/NA). Children aged from 13 to 24 months demonstrated notable difficulty in production questions. Children aged >24 months exhibited positive responses across most categories.

Figure 4 depicts the results of questions categorized by language development areas. The sensory reception category was evaluated only for children aged up to 12 months to discard hearing issues. Children aged <12 months encountered more challenges in the production and pragmatics categories. Furthermore, there were no cases of hearing problems. The pragmatics category showed an equal balance between successes and failures. Conversely, children in the second year demonstrated notable difficulty in answering questions within the language production category, with a higher-than-average rate of incorrect responses. Other categories showed satisfactory performance.

Finally, children aged >24 months exhibited predominantly positive responses across most categories. Hence, it becomes evident that children aged <24 months exhibit a higher frequency of errors, attributable to the inherent variability in language acquisition during this developmental stage. Consequently, there is a pressing need for greater flexibility in language milestones and extending their coverage across months.

**Figure 5:**
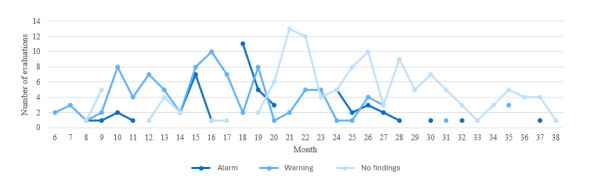
Distribution of suggestion type per month. Cases of children with language development issues were prevalent between months 15 and 27 (dark blue). The graphic reveals a gradual decline during the final months, with more typical development cases (pale blue).

As illustrated in Figure 5, this distribution reveals that instances of children with difficulty in language development are prevalent between months 15 and 27, gradually decreasing in the final months until 3 years of age.

In contrast, the evaluations were analyzed considering the control questions asked and the correlation between language development and the child’s date of birth. In Madrid’s nursery school classes, children were grouped based on their birth year. Consequently, at the beginning of the school year in September, a significant age gap existed between children born in the first quarter and those born in the last months. The latter group comprised the youngest in the class, resulting in a notable developmental difference between them.

**Figure 6:**
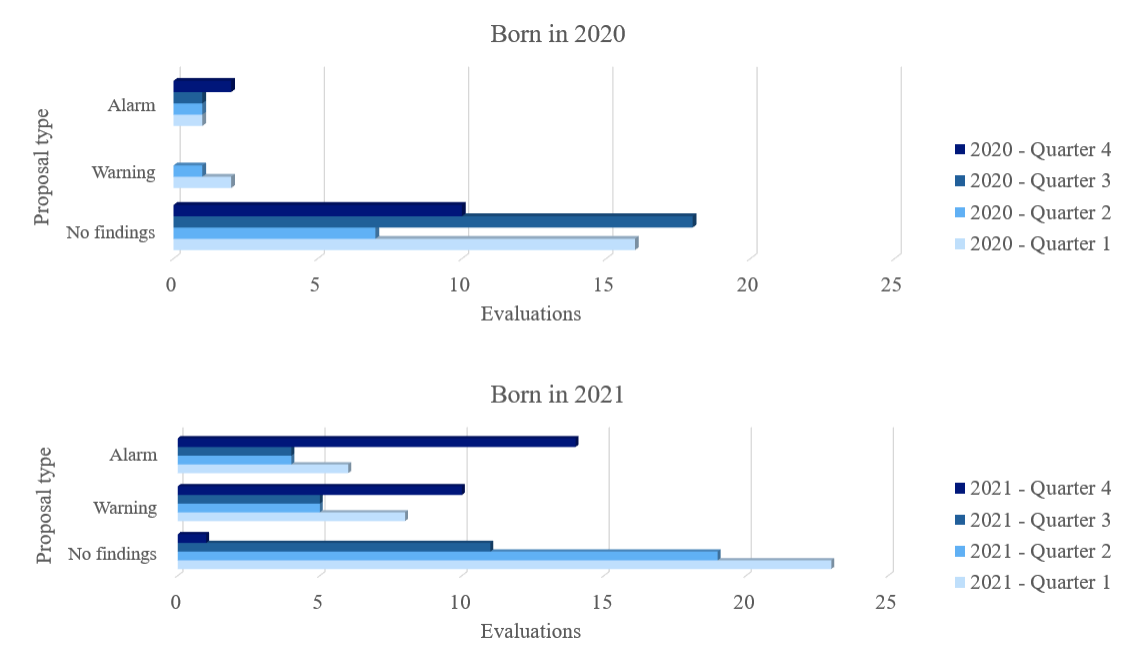
Assessment results by school quarter and suggestion type. For children born in 2020 (age between 25 and 36 months) alarm cases were more prevalent among children from quarter 4. However, these results are tempered by many more typical development cases at the same age. By contrast, for children born in 2021, the youngest children (quarter 4) tended to have alarm suggestions using the Gades platform.

The assessment outcomes for the youngest children in each class are presented in Figure 6. The youngest children were defined as those born from October to December 2020 for the age range of 25 to 36 months and from October to December 2021 for the age range of 13 to 24 months. This categorization was not applicable for children aged <12 months, as we did not have children from the last trimester. Hence, upon comparing the alarm for children born in 2020 and those born in 2021, it consistently appeared higher for those in the previous quarter. The youngest children tend to yield poorer results using Gades, as language development differs.

The results of the assessment outcomes in relation to the control questions can be observed in Table 6. It is noteworthy that the 2 children who disclosed a family history of other NDD in the control questions were identified as cases with difficulties in language development (ie, SLD or DLD cases). Among cases with reported issues during pregnancy (ie, perinatal, prenatal, or premature), a notable percentage resulted in difficulties in language development (9/13, 69% including warnings). In the case of preterm children, only 8 (3.7%) of the 218 cases were identified as cases with difficulties in language development. Only one of these preterm cases, a girl, was assessed as an alarm. The 3 cases of preterm boys without previous NDD demonstrated typical development. The remaining alarms in boys corresponded to cases of prenatal (3/218, 1.4%) and perinatal (4/218, 1.8%) risks. A similar scenario extended to bilingual cases, where only 1 child exhibited an alarm of language development difficulties.

**Table 6:** The child’s evaluations were distributed by control information, gender, and suggestion typea (N=218).

Gender and evaluation			Birth complications, n (%)					Family history, n (%)					Bilingual cases, n (%)		
			Center 1		Center 2		Center 1			Center 2		Center 1			Center 2
**Female**															
	Alarm	0 (0)		0 (0)		0 (0)			0 (0)		0 (0)			0 (0)	
	Warning	3 (1.4)		0 (0)		0 (0)			0 (0)		3 (1.4)			0 (0)	
	Typical development	1 (0.5)		0 (0)		0 (0)			0 (0)		4 (1.8)			0 (0)	
**Male**															
	Alarm	4 (1.8)		2 (0.9)		1 (0.5)			1 (0.5)		1 (0.5)			0 (0)	
	Warning	0 (0)		0 (0)		0 (0)			0 (0)		0 (0)			0 (0)	
	Typical development	3 (1.4)		0 (0)		0 (0)			0 (0)		2 (0.9)			0 (0)	

^a^Among children with birth complications, 69% (9/13) of them showed language development difficulties. Those with a family history of neurodevelopmental disorder also faced challenges, while only 1 bilingual child showed an alarm.

### Gades’ Performance

**Figure 7:**
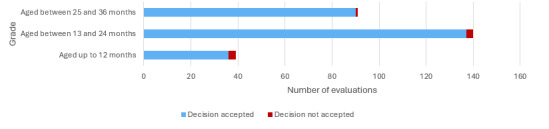
Gades acceptance per grade. Out of 270 evaluations, only 7 proposals were declined (ie, 3 for evaluations of the first and second grades and 1 for evaluations in children aged >24 months). Even so, the acceptance of Gades remains above 90% in all courses.

Gades’ performance is assessed by the extent to which the system’s decisions align with the perspectives of evaluators who support its recommendations. If there was disagreement with a recommendation, evaluators were prompted to justify dissent. Evaluators based their agreement or disagreement with the system’s decision on their understanding of the child, considering factors such as age, family environment, and communicative intent. According to the results, Gades exhibited an accuracy rate of 97.4% (263/270). Out of 270 evaluations, only 7 (2.6%) suggestions were declined by evaluators. Figure 7 shows a higher disagreement rate with the system’s decisions in children aged <24 months. Specifically, the accuracy rate was 92.3% up to 12 months, compared to 97.8% in the second grade (aged between 13 and 24 months) and 98.9% in the third grade (aged between 25 and 36 months).

Considering the total of 270 assessments conducted, including those where children were evaluated twice, the accuracy of Gades’ decisions is depicted in Table 7. Among the suggestions provided by the Gades system, where evaluators disagreed (7/270, 2.6%), some suggested referring the child to early intervention for a specialized assessment (5/270, 1.9%).

**Table 7:** Gades’ accuracy by the suggestion typea.

Suggestion description	Accepted suggestion, n (%)	
	No (n=7)	Yes (n=263)
Refer to early childhood care	5 (1.9)	45 (16.7)
Typical development	0 (0)	125 (46.3)
Repeat assessment (within 2 mo)	2 (0.74)	29 (10.7)
Repeat assessment (within 3 mo)	0 (0)	44 (16.3)
Repeat assessment (within 1 mo)	0 (0)	20 (7.4)

^a^Evaluators did not agree with Gades’ suggestion in 2.6% (7/270) of evaluations. Most of these unaccepted suggestions were for the most severe cases identified by Gades (“refer to early childhood care”). Gades exhibit an accuracy rate of 97.41%.

When the evaluator disagreed with the Gades’ decision, they were required to provide a comment stating the reason for nonacceptance. Thus, several reasons explain why the evaluators did not accept Gades’ decision (Table 8). In 4 instances (ie, for months 10, 12, 15, and 18), they noted that the questions were not tailored to the child’s developmental stage. In 2 cases from 18 months, the evaluator rejected the suggestion. Moreover, in case “The questions do not align with the child’s developmental stage,” the assessor disagreed with the suggestion and conducted an immediate reassessment. This subsequent assessment issued a warning (ie, to repeat the evaluation in the next months). In addition, in 2 cases involving 2 boys, the evaluators intended to continue observing the child’s progress as they had identified other communicative skills. Finally, there was no disagreement for typical development cases.

**Table 8:** Evaluator’s reasons for rejecting the final suggestion from Gadesa.

Reason			Children’s gender		Evaluation month
**Refer to early childhood care**					
	“We will monitor the situation closely. If necessary, we will consult the Early Assessment Team for guidance.”	Male		26	
	“There are alternative forms of communication besides verbal language, and he demonstrates proficiency in them. We will observe his development and see how it progresses.”	Male		18	
	“The questions do not align with the child’s developmental stage.”	Female		10	
	“The questions do not align with the child’s developmental stage.”	Male		10	
	“The questions do not align with the child’s developmental stage.”	Female		15	
**Repeat assessment (within 2 mo)**					
	“The questions do not align with the child’s developmental stage.”	Female		12	
	“We consider that it is not necessary at this stage.”	Male		18	

^a^The suggestions that were not accepted relate to “refer to early childhood care and repeat assessment (within 2 months)”. Most of the reasons were that the assessment conducted by Gades was not aligned with the child’s developmental stage.

### Overview of the Obtained Functional Results

Numerous interviews were conducted with the evaluation staff, and they completed a web-based form assessing the usefulness and practicality of the Gades tool in uncontrolled educational settings, following the guidelines outlined in the referenced study [41]. None of the evaluators had previous experience using this specific platform. It was noted that preschool educators and professionals in early childhood educational and psychopedagogical centers found Gades highly acceptable. Most (18/24, 75%) of the evaluators found the system easy to use, although it required dedicated time. They experienced occasional technological issues such as student search processes or time sessions. A quarter felt previous technological knowledge was necessary.

The educators used an observational methodology during the school day to respond to the form’s questions, considering it noninvasive and conducive to respectful support within an appropriate environment for the child. They suggested that implementing the Gades assessment during the first term of the school year, particularly during the adaptation period, could be challenging due to their limited familiarity with the child. In addition, they proposed conducting evaluations every 3 months for cases initially indicating “typical development” to ensure ongoing progress.

While control questions were valuable for accurate results from the Gades system, access to certain information, particularly related to birth difficulties, was limited for many children. Regarding the comprehension and appropriateness of the KB questions, educators encountered comprehension difficulties in only 2 out of the 108 questions, which they resolved by consulting the Gades’ technical staff. They noted that some questions were unsuitable for the child’s developmental stage. They highlighted the absence of questions about family context, communication dynamics, and disruptive behaviors in the second and third years.

Regarding recommendations, 75% (18/24) of the evaluators reported that they would suggest Gades to other nursery schools, emphasizing the need for previous DLD training to observe children effectively. They reported high knowledge acquisition and insight into their students’ language development. For instance, they had observed that girls aged <12 months initiated communication earlier and more fluently.

## Discussion

### Principal Findings

This study’s core findings underscore the Gades platform’s effectiveness as a robust tool for screening language disorders in preschool environments, successfully identifying language difficulties in 19.7% (43/218) of the children assessed. This rate, with a notable prevalence in boys (29/218, 13.3%) compared to girls (14/218, 6.4%), is significantly higher than the traditional prevalence estimates of 2% to 7% for DLDs [8,17,21,23,43]. This discrepancy suggests that Gades may be particularly sensitive to early signs of language difficulties or may also capture cases of SLD. As a screening tool rather than a diagnostic system, Gades aims to identify children at higher risk for DLD or SLD, which might lead to elevated detection rates compared to studies focused strictly on diagnosis. Comprehensive assessments by specialists at early intervention centers are required for a definitive diagnosis, highlighting the necessity of further evaluation following initial screening outcomes.

The study validated Gades’ technical and functional capabilities in uncontrolled educational settings without direct intervention by an NSLT. The platform’s utility in guiding nonexpert users, such as preschool teachers, through the screening process was particularly noteworthy. Teachers could use Gades effectively to observe and assess language development, reflecting its potential to empower educators with limited specialized training in language disorders.

Moreover, Gades demonstrated a high accuracy rate (97.41%) in aligning with the educators’ assessments, underscoring its reliability and the robustness of its underlying algorithms and KB. The discrepancies between the system’s recommendations and the educators’ judgments were minimal, indicating a high level of agreement and trust in the platform’s diagnostic suggestions.

The findings also illuminated specific age ranges (between 15 and 27 months) where language difficulties are most prevalent, reinforcing the importance of targeted early intervention during this critical developmental period. The ability of Gades to adapt its screening approach based on the child’s age and specific educational context was a key factor in its effectiveness.

These promising results suggest that integrating Gades into regular preschool assessments could significantly enhance the early detection of language disorders, potentially leading to more timely and effective interventions.

### Extending Detection to Natural Environments

This study highlights the importance of checking for developmental disorders similar to DLD in natural settings, such as preschools, where children naturally spend much time. DLD, similar to autism and ADHD, is among the most common developmental challenges but often goes unrecognized because its symptoms may resemble simple speech delays or overlap with other developmental disorders, leading to delayed diagnoses. Equipping educators, teachers, and school professionals with the right knowledge and support is crucial because they can spot early signs of these disorders. Early detection, significantly enhanced through collaboration with a multidisciplinary team, is key to improving a child’s prospects for developing language skills effectively.

### Applicability and Scalability

The Gades platform is designed to be versatile and scalable, making it a valuable tool for educators worldwide, especially in regions with limited access to specialized language disorder services. This flexibility is crucial for adapting to diverse linguistic and cultural environments across global educational systems. To effectively implement Gades in these diverse settings, developing training programs sensitive to local languages and educational standards is essential. These programs should equip educators with the skills to use the platform effectively and to recognize early signs of language disorders, even without specialized training.

Considering the technological limitations in different regions, Gades should be designed to operate in low-bandwidth environments and comply with local data protection regulations. This will make the platform accessible and trustworthy. Enhancing the platform’s database to support multiple languages and reflect cultural specifics can make Gades a more inclusive tool that accurately reflects diverse child development patterns.

Practical implementation strategies include collaborating with local educational authorities to incorporate Gades into routine screening processes. This might involve tailoring the platform to meet local developmental benchmarks and language nuances. Partnering with local educational institutions can provide continuous support and feedback, helping to refine the platform.

Gades could offer versions that range from sophisticated, high-tech applications to more basic, web-accessible formats to accommodate varying technological capabilities. Establishing a community of practice among Gades users would encourage sharing best practices and provide essential peer support, enhancing the platform’s overall effectiveness and user experience.

### Limitations

One significant limitation of the Gades system is its reliance on the accuracy of the input provided by educators, who may not have specialized training in identifying language disorders. This dependency can introduce biases or inaccuracies in the screening process, as nonexpert interpretations of language development may vary widely, affecting the reliability of the outcomes. To mitigate these potential biases, it is essential to implement comprehensive training programs for educators using the platform. These programs should focus on familiarizing them with common language development milestones and the specific signs of language disorders. In addition, incorporating automated prompts and guidelines within the Gades system can help standardize recorded observations, reducing subjective interpretation errors.

Further refining the interface and feedback mechanisms of Gades can also enhance the accuracy of the data collected. For instance, the platform could include illustrative examples or short training videos on typical versus atypical language behaviors to guide educators’ assessments before they input data. Regular updates and calibrations based on user feedback and new research in language development could further improve the system’s precision and adaptability to real-world educational environments.

The study’s limitations extend beyond the technical aspects of the Gades platform to include concerns about the sample size and the uncontrolled implementation settings. The small sample size may not adequately represent the broader population of preschool children, limiting the generalizability of the findings. In addition, while providing real-world insights into the use of Gades, the uncontrolled setting might introduce variability in how the platform is used across different environments, potentially influencing the consistency and applicability of the results. These factors could lead to higher variability in outcomes, which might not fully indicate the platform’s efficacy under different or more controlled conditions.

### Future Directions

Future research should include larger and more diverse populations to address the limitations mentioned to ensure that findings are robust and widely applicable. Controlled trials, where variables can be more tightly managed, would also help understand the platform’s effectiveness across varied educational settings and populations. This would allow for more reliable validation of the platform’s capabilities and identify specific conditions under which it performs best, guiding more targeted improvements.

To enhance Gades’ effectiveness and reliability, it is imperative to refine the tool by expanding the KB to better differentiate between DLD and SLD at earlier stages. Adapting the system to align more closely with the actual ages of children rather than their academic year will provide more accurate assessments. Implementing educator training modules will equip them to effectively recognize early signs of language difficulties. Future versions of Gades should include a nuanced approach for assessing the youngest children in the class, considering their specific developmental stages and potential delays without prematurely recommending specialist evaluations. These improvements will ensure that Gades supports early detection and integrates smoothly into regular preschool assessments, potentially enhancing the effectiveness of interventions for language disorders.

### Conclusions

This study confirms that DLD remains a commonly overlooked condition with significant variability and potential overlaps with other developmental disorders. Importantly, our findings reinforce the critical nature of age-specific screenings, with the most alarms for language difficulties arising in second-year grades, supporting the hypothesis that this period is crucial for detecting DLD risk factors. Despite the conservative and generic screening process, which identified language difficulties in 19% of cases—higher than the 7% prevalence typically noted in other studies—some of these instances may represent SLD. This suggests a need for refining language milestones within the Gades system to more effectively distinguish between DLD and SLD.

The qualitative outcomes from the Gades platform demonstrate its value in designing tailored educational programs and therapeutic interventions. By integrating such tools without the need for direct support from NSLT, Gades facilitates a deeper understanding of individual language development patterns. This insight enables educators, particularly those without specialized training in language disorders, to identify and address developmental challenges more effectively and earlier. The platform has been well-received in terms of usability and practicality, enhancing educators’ ability to actively monitor and support language development.

Moreover, implementing Gades has provided substantial learning opportunities for preschool teachers. Educators with no previous expertise in DLD have gained significant knowledge and skills in informal screening processes, which has had a transformative impact on their ability to recognize and respond to language development issues. This empowerment of teachers underscores the potential of Gades to serve as a foundational tool in early education settings, enhancing early detection and intervention strategies.

To extend the utility of Gades beyond this study context, it is crucial to provide practical recommendations for its implementation in various educational settings. Tailoring the platform to accommodate different regional educational standards and linguistic backgrounds can maximize its effectiveness and reach. In addition, the successful deployment of Gades is contingent upon comprehensive training programs for educators. These programs should focus on enhancing understanding of language milestones, screening techniques, and the specific functionalities of the Gades platform. Effective training could be implemented through web-based modules, workshops, and ongoing support systems to ensure educators can proficiently use the tool.

In conclusion, the Gades platform represents a significant advancement in the field of educational technology for screening language disorders. By facilitating detailed observation and reporting of language development, Gades supports educators in their daily interactions with children and contributes to a broader strategy for addressing DLDs in early childhood education. The adoption of such tools, accompanied by adequate training and tailored implementation strategies, holds the promise of significantly improving outcomes for children at risk of DLD and SLD across diverse educational landscapes.

## Abbreviations

ADHD: attention-deficit/hyperactivity disorder
DLD: developmental language disorder
DSM-5: Diagnostic and Statistical Manual of Mental Disorders, Fifth Edition
KB: knowledge base
NDD: neurodevelopmental disorder
NSLT: nursery school language therapist
SLD: simple language delay

## Data Availability

The datasets generated and analyzed during this study are available from the corresponding author upon reasonable request.
